# Evidence of anticipatory forest use behaviours under policy introduction: a systematic map protocol

**DOI:** 10.1186/s13750-023-00307-0

**Published:** 2023-09-26

**Authors:** Jorge Claudio Llopis, Neal Robert Haddaway, Nurzhan Omirbek, Blake Alexander Simmons, Rachael Garrett, Julia Patricia Gordon Jones

**Affiliations:** 1 School of Natural Sciences, Bangor University, Bangor, UK; 2 Interdisciplinary Centre for Conservation Science, University of Oxford, Oxford, UK; 3 Centre for Development and Environment, University of Bern, Bern, Switzerland; 4 Leibniz Centre for Agricultural Landscape Research, Müncheberg, Germany; 5 Africa Centre for Evidence, University of Johannesburg, Johannesburg, South Africa; 6 Tampa Bay Estuary Program, St. Petersburg, FL USA; 7 Department of Geography and Conservation Research Institute, University of Cambridge, Cambridge, UK

**Keywords:** Forest conservation, Habitat conservation, Biodiversity, Area-based approaches, Climate change, Environmental policy, Human behaviour, Unintended outcomes

## Abstract

**Background:**

Forest conservation is a major global policy goal, due to the role forests play in climate change mitigation and biodiversity conservation. It is well recognized that the introduction of policies, whether aimed at forest conservation or with other objectives, has the potential to trigger unintended outcomes, such as displacement or leakage, which can undermine policy objectives. However, a set of outcomes that has escaped detailed scrutiny are anticipatory forest use behaviours, emerging when forest stakeholders anticipate policy implementation, deploying for example pre-emptive forest clearing, resulting in detrimental environmental outcomes. Lack of understanding of the extent and sectorial scope of these behaviours prevents us from devising strategies to address their potential detrimental consequences.

**Methods:**

This protocol presents the methodology that will be followed to conduct a systematic map to identify, compile, review and describe the evidence available on anticipatory forest use behaviours in the context of policy introduction around the world. We will use two complementary search strategies, which we have tested before submitting this protocol. First, a systematic bibliographic search, and second, a citation chase approach. We will include articles based on a pre-defined set of criteria defined according to a Population, Intervention and Outcome (i.e. PIO) design. To support identification of knowledge gaps and clusters, we will report results of the systematic map in a narrative synthesis, an evidence atlas and other visualisations.

**Supplementary Information:**

The online version contains supplementary material available at 10.1186/s13750-023-00307-0.

## Background

Sound forest conservation policy is needed to tackle the combined challenges of climate change and biodiversity decline [8], for which standing forests play a key role [6, 54]. The forest conservation agenda is receiving attention at the highest level of international policy making; for example at the United Nations Climate Conference of Parties 26, leaders from 141 countries pledged to stop deforestation by 2030 [52]. However, the introduction of policy, whether aimed at forest conservation or other objectives, has the potential to trigger unexpected outcomes. In forest conservation approaches, such as the establishment of terrestrial protected areas, one of the best studied unexpected outcomes is displacement effects (also known as leakage), where avoided deforestation is spatially displaced elsewhere [43]. The implications of these unintended outcomes of forest conservation policy are well understood and considered by policy makers [5, 46]. However, a set of unintended outcomes of forest conservation policy introduction that has so far escaped detailed scrutiny is that of anticipatory behaviours, where forest edge residents, landholders or forest users change behaviour in advance of policy implementation.

Anticipatory behaviours are well-known in other policy contexts, and have been investigated extensively in the economics literature, for example in the case of taxation [1, 7] or health policy reform [3], or housing markets under urban development dynamics [27]. In the case of environmental policy introduction, anticipatory behaviours might result in what has been labelled in the climate policy sector the Green Paradox [49], where introduction of tighter regulation results in increasing pre-emptive extraction of fossil fuels [53], or a policy aimed at reducing pollution initially increases it [29]. In the context of biodiversity conservation, anticipatory behaviours have been demonstrated in the case of international wildlife trade, with traders anticipating species-wide trade bans by increasing commercial transactions of the species targeted before the ban is implemented [38, 45]. A comparable phenomenon has been observed in cases of marine reserve establishment [37], or the management of fishing quotas [26]: in both cases with fishers anticipating the implementation of the policy change by increasing their short-term fishing efforts.

**Table 1:** Examples of anticipatory forest use behaviours in contexts of terrestrial biodiversity conservation policy introduction, and in policies with the potential to affect forests (e.g. agricultural development and sustainable agricultural production)

Sector	Intervention	Study examples	Location	Anticipatory behaviour
Terrestrial biodiversity conservation	Habitat conversion regulation	Stroup [50]	North Carolina, United States	Timber harvest rotation shortening
		List et al. [32]	Arizona, United States	Pre-emptive forest clearing
		Simmons et al. [48]	Queensland, Australia	Pre-emptive forest clearing
		Seghezzo et al. [47]	Salta, Northern Argentina	Pre-emptive forest clearing
	Terrestrial protected area establishment	Keller [30] Llopis et al. [33]	Northeastern Madagascar	Pre-emptive forest clearing
	Payment for Ecosystem Services scheme	Fiorini et al. [15]	Rio de Janeiro State, Southern Brazil	Forest regrowth suppression
Agricultural development	Land registration program	Middleton [39]	Southern Madagascar	Pre-emptive forest clearing
		Grimsditch and Schoenberger [16]	Cambodia	Pre-emptive forest clearing
		Wren-Lewis et al. [56]	Benin	No anticipatory behaviours found
	Agrarian reform	Alston et al. [4] Aldrich et al. [2]	Pará, Brazilian Amazon	Pre-emptive forest clearing
Sustainable agricultural production	Certification for sustainable production of palm oil	Carlson et al. [9]	Sumatra and Kalimantan, Indonesia	Pre-emptive forest clearing

References are selected from our initial article benchmark (Additional file 1)

**Fig. 1:**
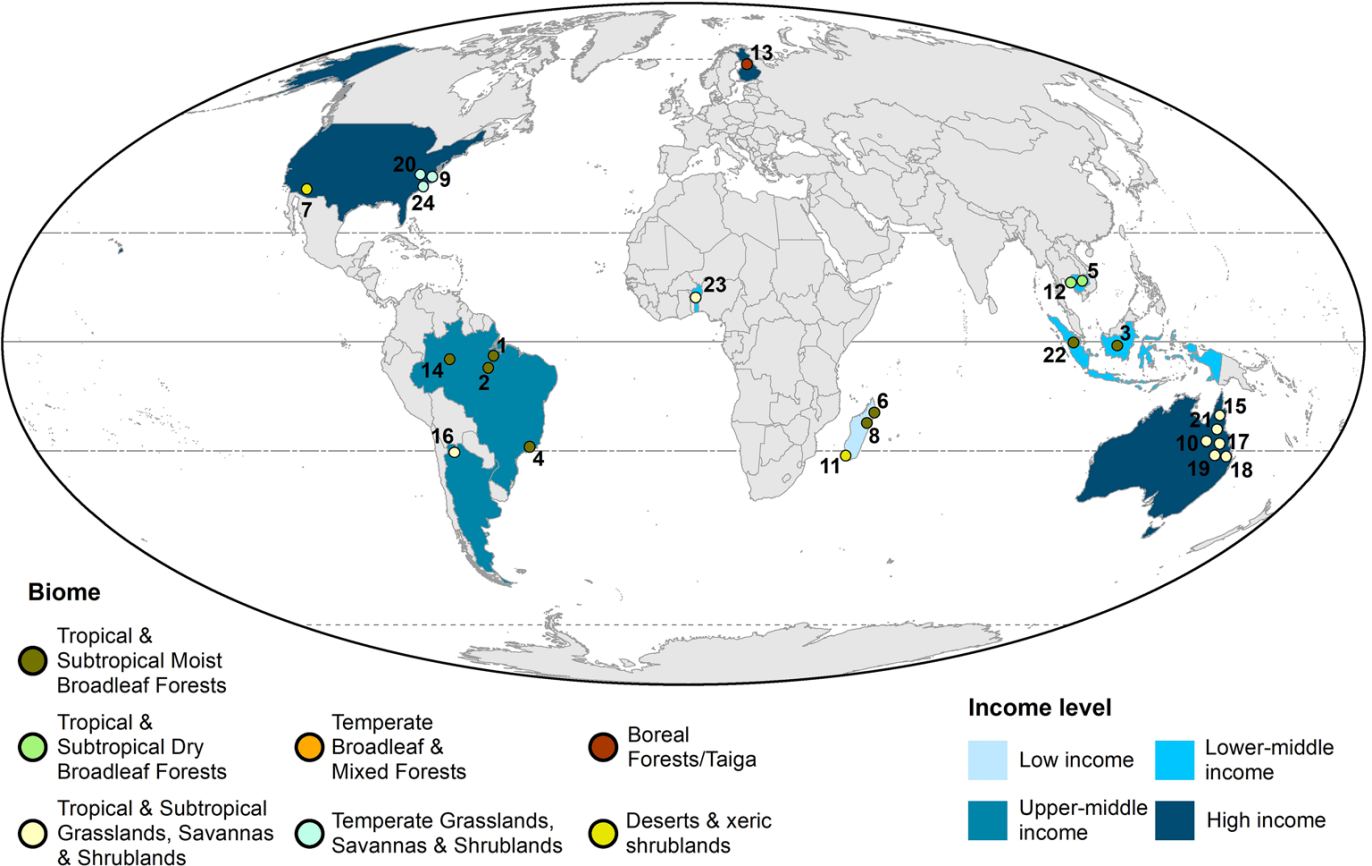
Location of initial benchmark articles (Additional file 1). For visualization purposes, location is provided approximately, see Additional file 2 for more information. Biome classification based on Dinerstein et al. [14]. Income level classification based on World Bank [55]

In the context of introduction of policy directly aimed at forest conservation, several cases of anticipatory behaviours have been reported (Table 1) across biomes and country income levels (Fig. 1). A classic example is the case of the Endangered Species Act introduced in the United States in the 1970s and updated subsequently to include new species for habitat conservation. To avoid restrictive land use regulations, in several cases landowners pre-emptively destroyed an endangered species’ habitat within their property between the moment the species was listed for protection and the prohibition to damage its habitat was actually enforced  [32, 35, 57]. A comparable situation emerged in Queensland, Australia, with landholders ‘panic clearing’ native forests and woodlands in advance of the implementation of and amendments to vegetation management regulations restricting land clearing from the 1990s to the 2010s [44, 48]. In the case of area-based forest conservation interventions, such as establishment of terrestrial protected areas, a related phenomenon was reported in Northeastern Madagascar, with forest edge populations increasing their forest clearing efforts before the protected area was in place, in an attempt to secure agricultural land [30, 33]. Such pre-emptive forest clearing was also found in voluntary participation in sustainability certification of agricultural commodities production, such as palm oil, in Sumatra and Kalimantan, Indonesia [9].

Policy changes that are not directly concerned with forest can also result in anticipatory behaviours negatively affecting forest cover or condition. For example, when a land registration initiative is being initiated, local populations might clear forest to access agricultural land in the expectation they will obtain a statutory recognition for that parcel, such as a title or certificate. This was found to have happened in the case of a nation-wide program in Cambodia [16], and likely also in Southern Madagascar [39], although land titling does not always result in such an effect [56].

These examples suggest that anticipatory forest use behaviours might be a common response to policy introduction, potentially undermining the intervention’s objectives. However, no systematic evidence synthesis has been conducted on the phenomenon. To fill in this knowledge gap, we will develop a systematic map to compile and synthesize the global evidence of the emergence of anticipatory forest use behaviours under policy introduction contexts detrimentally affecting forest cover or condition. Given that this is the first such evidence synthesis effort, a systematic map is the most appropriate approach because of uncertainty about the extent to which these behaviours have been studied and reported, as well as likely wide variation in the type and quality of the reports in the literature. Systematically mapping the evidence available on these behaviours might benefit environmental policy and management by providing an accessible and easy to consult repository of cases [18], helping increase decision-maker’s awareness of the risk of policies triggering such unintended behaviours, and may stimulate more formal study of the phenomenon. The evidence on anticipatory forest use behaviours under policy introduction already listed above has been compiled into an initial benchmark list (Fig. 1; Table 1, Additional file 1), which has helped to inform and test our search strategies (see below). This evidence was gathered by the authors over recent years, and expanded through a call for evidence from experts released by the first author on the social media network Twitter in April 2022.

## Objective of this systematic map

The objective of this systematic map is to assemble and map the current state of evidence describing anticipatory forest use behaviours as a result of policy introduction, focusing on behaviours that detrimentally affect forest cover or condition.

### Primary question

The primary research question that will guide the systematic map is: *What is the state of the evidence on anticipatory forest use behaviours in the context of policy introduction?*

This question was defined by the reviewing team after consultation with relevant stakeholders consulted prior to submitting this protocol for review.

#### Elements of the primary question

This systematic map will follow a population-intervention-outcome (P.I.O) framework, with the question elements being the following.

#### Population

We focus on global forests as the population of interest. The global nature of this systematic map is justified on the basis of the evidence on anticipatory forest use behaviours gathered so far (Additional file 1), which have been found across country income levels and biomes (Fig. 1).

#### Intervention

The interventions of interest in this systematic map are the policies prompting anticipatory forest use behaviours that detrimentally affect forest cover or condition.

#### Outcome

The outcome of interest in this systematic map is the anticipatory forest use behaviour the policy intervention triggered, that would have been regulated by the intervention, and that detrimentally affects forest cover or condition.

## Methods

This evidence synthesis will follow guidance on systematic maps [28, 41], and will conform with ROSES reporting standards [22] ﻿﻿﻿﻿(Additional file 3)﻿. This protocol has been compiled following the Collaboration for Environmental Evidence guidelines 8 [13].

### Stakeholder engagement

Given the applied nature of this research, and the urgency to transfer the knowledge to relevant stakeholders, we will follow best practice guidance on stakeholder engagement [18]. We have identified a group of stakeholders with expertise in each of the broad policy areas we have found to trigger anticipatory forest use behaviour so far. Concretely, we are consulting with an expert on terrestrial protected areas establishment and management, an expert on land tenure specializing in land rights formalization processes, and an expert on sustainable agricultural production at the forest frontier. Expert is understood here as an individual with practical experience in development or implementation of environmental policy or an established researcher who has focused much of his or her scientific career on the intervention we are interested in. We have shared the protocol with these stakeholders in parallel to the review process in the Environmental Evidence journal, and their input was implemented at the same time that we addressed the reviewers’ comments on the protocol. The author team will also request comments about the clarity of the final report from these stakeholders once the synthesis is conducted.

Our review team is also highly interdisciplinary with links to relevant areas of policy. We have one specialist in global land use policy (RG), an expert on protected areas (JPGJ), and an expert on pre-emptive forest clearing behaviours (BAS), as well as an expert on evidence syntheses (NRH).

### Searches

#### Search strategy

For this systematic map we will follow two complementary search strategies: a bibliographic search, and a citation chasing strategy.

##### Strategy A—bibliographic search

We will conduct a comprehensive bibliographic search through four bibliographic and two dissertation platforms (Table 2), one search engine (Google Scholar) and 67 organisational websites (Additional file 4). We have developed a search string (Table 3) based on the terms employed by the initial benchmark articles to describe anticipatory forest use behaviours, including relevant synonyms to those terms, as well as extracting terms used to describe forest ecosystems in other systematic map protocols (e.g. [11]). We have included in the search string the population element of the research question (e.g. forest, woodlands, etc.), as well as the outcome element, separated into the forest use behaviour undertaken (e.g. deforestation, clearing, expansion, etc.) and terms which seek to capture the anticipatory character of the behaviour (e.g. pre-emptive, anticipatory, unprecedented, etc.). We have not included the intervention element given that, after testing different search string including it (Additional file 5), we realised that its inclusion produced a heavily inefficient string that resulted in insufficient specificity, thus returning a large number of results not relevant to the research question. The search string was tested and refined as explained in Additional file 5, to keep a balance between sensitivity and retrieving a manageable amount of articles. On a scoping search we conducted on 19/06/2023 in Web of Science Core Collection and Scopus on title, abstract and keywords (respectively TOPIC and TITLE-ABS-KEY), our search string returned 7407 and 9695 results respectively.

We will search in the bibliographic databases presented in Table 2. Some collections contained on certain platforms do not allow exporting results, so we will exclude those databases for our search. For the dissertation databases, we will restrict the search to items for which full text is available in the respective databases. For searching Google Scholar we will adapt the search string, and use the “Publish or Perish” [25] tool, including the first 300 results as recommended when using Google Scholar for this task [19].

**Table 2:** Bibliographic and dissertation databases, and web-based search engine to be used

Type	Platforms*	Database	Web URL
Bibliographic database	EBSCOhost	GreenFILE, Library, Information Science & Technology Abstracts, and MEDLINE	https://web.a.ebscohost.com
	ProQuest Core Databases	SciTech Premium Collection; Social Science Premium Collection	https://www.proquest.com
	Scopus	Scopus	https://www.scopus.com
	Web of Science core collection	SCI-EXPANDED; SSCI; AHCI; CPCI-S; CPCI-SSH; ESCI	https://www.webofscience.com/wos/woscc/advanced-search
Dissertation database	EBSCOhost	Open Dissertations	https://biblioboard.com/opendissertations
	ProQuest	Dissertations & Theses Global	https://www.proquest.com
Web-based search engine	Google Scholar	Google Scholar	https://scholar.google.com

*Searches will be conducted using subscriptions of Bangor University, UK

The 67 organisational websites selected (Additional file 4) were chosen based on the research team’s expertise, complemented by retrieving the list of organisational websites employed in other forest-related systematic map protocols [10]. Where the publications page does not provide an advanced search function, we will first use the search string for the population element (Table 3) and look into the first 50 records of each website. In the case of websites of forest-related organisations, we will instead use the string for the outcome element. Where the search options include searching by topics, only those most relevant to the objective of this systematic map will be searched into (e.g. forestry, rural livelihoods, etc.). We will not investigate scientific articles contained within organisational websites, as these will be captured by our search of bibliographic databases. Results from searching into organisational websites will be downloaded and merged with results from the bibliographic search when possible. In cases where downloading results in a .csv format is not possible, we will systematically download results using either the Grey Literature Search Recorder app (https://www.eshackathon.org/software/grey-lit-reporter.html), the greylitsearcher Shiny app [17], Publish and Perish [25], or the Data Miner Chrome extension (https://dataminer.io). If that is not possible in specific websites, we will screen the items online, and relevant results will be exported.

**Table 3:** List of search terms in English language, using Web of Science syntax

Substring	Search terms
Population	(tree* OR *forest* OR woodland* OR “wood*-land” OR habitat* OR vegetation OR timber OR canopy OR mangrove* OR savanna* OR cerrado OR “caatinga”)
	AND
Outcome (forest use behaviour)	(deforest* OR clear* OR destruct* OR extracti* OR conversion OR convert* OR destroy* OR vanish* OR fell* OR cut* OR expansion OR expand* OR “land use change” OR “land-use change” OR “land cover change” OR “land-cover change” OR “land change” OR “land-change” OR replac* OR remov* OR harvest* OR logg* OR exploit* OR “land management”)
	NEAR/10
Outcome (character of behaviour)	(pre-empt* OR preempt* OR anticipat* OR prematur* OR contentious OR contended OR unprecedented OR panic OR exacerbat* OR accelerat* OR reinforc* OR rush* OR unplanned OR unpredict* OR unexpect* OR expectation* OR atypical* OR perverse* OR unintended OR spik* OR stimulat* OR preventive OR preventative OR paradox* OR undesir* OR violen* OR *incentiv* OR ambiguous)

After screening results, the articles (see *Article Screening* section below), the articles found to be relevant will be merged with those in the initial benchmark list (Additional file 1), to elaborate an extended benchmark list, on which we will apply the citation chase approach (see below).

##### Strategy B—citation chasing

We will apply a citation chase strategy on the extended article benchmark produced from the bibliographic search. We will conduct both, backward citation chase (i.e. gathering the references cited by the benchmark articles), and forward citation chase (i.e. retrieving the articles citing those in the benchmark). When backward citation chasing on books (edited or otherwise), dissertations, and organisational reports, we will only explore references from the chapters or sections containing the information we are interested in (i.e. on anticipatory forest use behaviours). Second, we will remove results that are either newspaper articles, court hearings, government documents (e.g. including laws, decrees, official speeches, etc.), as well as results obviously not relevant to this study, such as references to datasets, methods, R packages, etc., and all results that are not in English language. Third, we will remove duplicates. And fourth, we will screen for relevance the remaining articles at title and abstract level (concurrently), and then proceed to full text screening those articles found relevant at title and abstract following the inclusion criteria listed below.

We tested the performance of the dedicated tool citationchaser [22], available as an R package and a Shiny app, on the initial article benchmark. For the backward citation chase we tested the performance of the citationchaser Shiny app against the same task conducted through Web of Science, Scopus and manually. Results (Additional file 6) indicate that citationchaser performed worse (it got fewer references from each benchmark article) than Web of Science or Scopus, while Scopus yielded nearly identical results to manually extracting the references. For the forward citation chase, we compared the performance of the citationchaser Shiny app in finding the articles citing those in the benchmark against the same task in Web of Science, Scopus and Google Scholar. We found that citationchaser performed better (it found more citations for the benchmark articles) than either Web of Science or Scopus, but worse than Google Scholar.

Based on these tests, we will therefore use Scopus for backward citation chasing; manually extracting references from those articles in the extended benchmark that are not found in that database (mostly those without a DOI), and those for which Scopus yielded fewer references than we manually counted in the benchmark article. For forward citation chase, we will use the citationchaser Shiny app, resorting to Scopus in the case citationchaser cannot find any citation for the specific article, and using as last option Google Scholar, which seems the appropriate sequence based on more in-depth tests conducted on using Google Scholar to obtain citations [36].

### Estimating comprehensiveness of the search

**Fig. 2:**
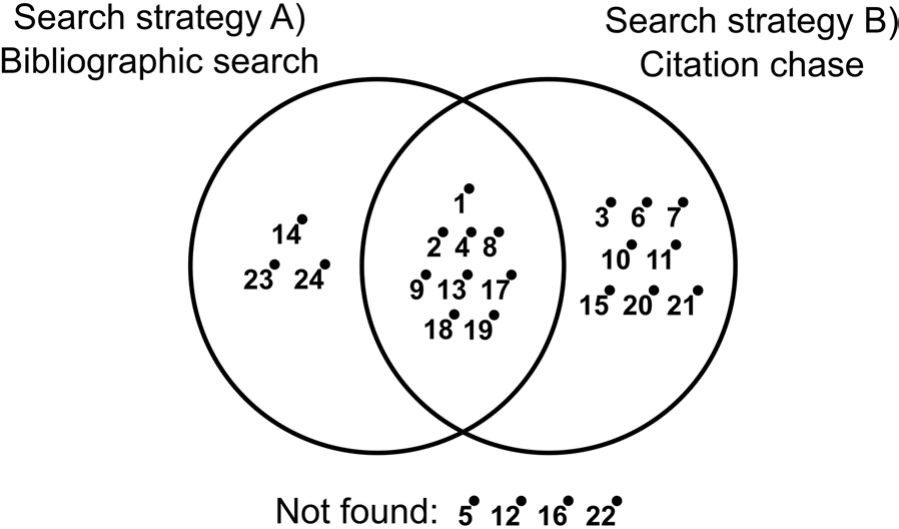
Comprehensiveness diagram showing the strength of each search strategy and their complementarity in finding the articles in the initial benchmark (Additional file 1)

To check how comprehensive our overall search approach is, we have tested the strength of the combined two search strategies at finding the articles in the initial benchmark (Fig. 2). The numbers presented are based on the search string test (Strategy A and Additional file 5) and the citation chase test (Strategy B and Additional file 6).

Citation chase is the strongest search strategy, finding 17 out of the 24 articles in the initial benchmark (Fig. 2). The bibliographic search managed to capture 12 benchmark articles. Together, the combined strategies captured 20 articles (83%). Of the benchmark articles that we could not detect, two were an organisational report [16], and a relatively old conference proceeding [51], both without a DOI and not indexed in any bibliographic dataset. The remaining two were Seghezzo et al. [47] and Milne [40] but which did not mention anticipatory forest use behaviours in title or abstract. We recognised that the search strategy is not perfect, although after extensive testing we believe our approach is as good as possible with the resources available.

### Article screening and study inclusion criteria

#### Screening strategy

We will retrieve title, abstract and all other information for all articles sought through search strategies A and B, import them into a reference manager software (i.e. Zotero or EndNote), and deduplicate results. Where different versions of the same article are found (e.g. a pre-print version and a definitive published version), we will keep the published version. Unique records will be then imported to an open access online tool designed for the management of systematic reviews and maps, either Rayyan [42] or CADIMA [31]. After the consistency check (see below), screening articles will be performed by at least two members of the review team independently, with the full dataset of records distributed among the reviewers.

#### Consistency checking

Checking consistency will be conducted across the screening process at both stages, i.e. title/abstract and full text. A random subset of 10% of the articles to be screened will be screened concurrently at title and abstract by all reviewers. We will then calculate the Cohen’s Kappa score [12], identify and discuss the reasons for disagreement article by article, and clarify the inclusion criteria. In the case the Kappa score does not reach a minimum of 0.6 in this first consistency check (indicating lack of consistency among reviewers), another 10% of articles will be then screened by all reviewers, and disagreements discussed. We will then calculate the corresponding Kappa score, again aiming at having a minimum of 0.6 to proceed to the next stage. In the case agreement between reviewers is still not achieved at this stage, we will repeat the procedure and check for consistency until agreement is reached. Once we have reached agreement among reviewers at the title/abstract screening stage, we will proceed to full text screening, conducting a similar consistency check.

#### Inclusion criteria

We will decide whether to include screened articles in the systematic map according to clear inclusion criteria. First we will check whether the article focuses on the appropriate Population, Intervention and Outcomes (see below for expanded definitions). When screening at title/abstract level for inclusion to full text screening, we will tend towards inclusion where there is uncertainty, given that in many occasions, the articles found reporting on anticipatory forest use behaviours at full text do not do so on title or abstract. Only articles in English will be included, given resource constraints. We do not have a date range criterion for inclusion of articles, given that this is the first evidence synthesis conducted on anticipatory forest use behaviours.

##### Relevant population(s)

The study mentions forest ecosystems or related concepts. Articles in the initial benchmark refer to forests in varied ways including ‘native vegetation’, ‘woodland’, or ‘habitat’ (see search string in Table 3). Given that most articles in the initial benchmark (Additional File 1) do not provide a definition of forest, providing such a definition is not an inclusion criterion. We will nonetheless compile definitions of forests from included articles whenever they are provided, and present them in the final systematic map report. We will not restrict inclusion by forest biome or country, given that the initial benchmark includes studies from a wide range of biomes and countries. However, included studies need to mention the place where the behaviour emerged, at least at the national level.

##### Relevant intervention(s)

The study mentions the policy that triggered the anticipatory forest use behaviour. We will include studies that provide evidence on any of the policies already found to encourage anticipatory behaviours, either with direct forest conservation objectives or otherwise (See initial article benchmark in Additional file 1), and also any other policy not yet captured in the benchmark articles. Given that we found anticipatory behaviours triggered by a wide range of policies (Table 1), and that the articles compiled in the initial benchmark (Additional file 1) are unlikely to capture the entire range of possible interventions triggering such behaviours, we do not define *a priori* the potential interventions. Policy in this systematic map is understood in a broad sense, including implementation of new laws, regulations or guidance, and changes or updates to existing policy. The policies of interest include global, national or sub-national policy instruments with a broad sectorial scope, including those with conservation objectives (e.g. establishment of terrestrial protected areas, species conservation regulations, REDD + projects), and those which might indirectly influence forest dynamics (e.g. land registration programs, agrarian reform processes, sustainability certification of agricultural commodities).

We refer to policy introduction as the entire process of policy development, including policy design, political discussions preceding implementation, announcement of the policy and actual implementation. We understand policy implementation as the cut-off date when the policy took effect, such as when a protected area was established, or a policy enacted.

To be included in the systematic map, the study needs to provide information about the date when the policy (or policy change) was implemented, or at least when the policy was or is expected to be implemented in the case it is not yet in place. We will also include studies where anticipatory behaviours were found to emerge in the case of policies that were planned but eventually were not implemented.

##### Relevant outcome(s)

The study describes anticipatory forest use behaviours, regardless of whether the term ‘anticipatory’ is used or not. The key inclusion criteria is that the study suggests anticipation of the policy as a likely explanation for the emergence of changes in land use which detrimentally affect forest cover or condition. These can include among others anticipatory behaviours that involve increases in the following forest cover dynamics: reduction of forest cover (e.g. clearing of forest for conversion to agriculture or other land use, clear cutting of timber), reduction of forest condition (e.g. selective logging, charcoal production) or others not yet identified in our benchmark.

Given that this is the first evidence synthesis on such type of complex behaviours, we will lean towards inclusion of all types of evidence on them. We will include articles where anticipatory forest use behaviours were explicitly expected to be emerge, but could not be detected (e.g. [56]), articles that provide anecdotal evidence on the behaviours, even if the article did not have them as the main focus of study (e.g. [30]), and articles where, the authors argue that they might be emerging and provide plausible mechanisms (e.g. [39]).

##### Relevant study design(s)

No study design types will be excluded during the screening stages. We will only include studies providing new evidence, and not those articles referring to secondary evidence (e.g. referring to other studies’ evidence). Exceptions to this rule will be made in the cases that the article screened reports evidence found in a type of source we are not screening, such as articles in languages others than English, government reports, books or newspaper articles. We will include articles where the primary data was collected elsewhere, but has not yet been reported.

### Reasons for exclusion

We will include in the final systematic map report a list with the studies excluded after full text screening, with the reasons for exclusion for each of them. Each study must meet all the inclusion criteria presented above to be considered relevant for this systematic map. We will thus exclude articles that do not provide information on each of our research question’s elements, i.e. they do not focus on forests (population) and on forest cover change (outcome-forest use behaviour), do not mention policy introduction (intervention), or do not mention anticipation (outcome-character of the behaviour). We will also exclude articles in languages other than English, as well as review articles, unless the evidence reported there is not available elsewhere.

### Data coding and extraction strategy

Data coding and extraction will be done to a large extent by the corresponding author (between 30% and 50% of the total included studies), as done in other systematic maps [34], who will then harmonize the way the data is extracted by the rest of the review team. For each included study, we will extract information on the variables presented in Table 4. One article can describe several studies, e.g. if the article is dealing with distinct locations, policy interventions or time periods. Where an article provides sufficient information to disaggregate into separate studies, we will extract and code information for studies separately. Also, several articles can refer to the same study, e.g. same location, policy and time period. In that case, and whenever the evidence provided is first hand, we will include all articles referring to the same study, classifying the study as a unique one, to make sure there are not duplicated studies in the final map. The metadata form has been tested on the 24 articles in the initial benchmark (Additional file 2).

**Table 4:** List of variables to code from the studies

Topic	Coding variable	Variable description
Bibliographic information	ID_article	Unique ID of the article
	ID_study	Unique ID of the study
	Study_short_title	Short string title for study
	Authors	List of authors
	Article_title	Article title
	Year	Article publication year
	Keywords	Article keywords as provided by authors
	Journal_publisher	Journal where the peer-reviewed article was published, book and publishing company where the book chapter appeared, organisation that published the report or working paper, or university where the dissertation’s degree was obtained.
	Document_type	Type of document: peer-reviewed article (e.g. commentary, opinion, full research, editorial, etc.), book chapter, dissertation (e.g. PhD, MSc, etc.), organisational report, conference proceedings, etc.
	DOI	Document DOI
Study location	Country	Country or region
	Locality	Site name describing the locality
	Scale	Geographical scale of the study, e.g.: i. Local ii. Subnational iii. National iv. International (e.g. more than one country)
	Biome	Biome where the study is located
	Latitude	Geographic latitude in decimal degrees
	Longitude	Geographic longitude in decimal degrees
	Coordinates_source	Source of latitude and longitude coordinates, e.g.: i. Provided by study ii. Imputed by reviewer (based on location on map, or location name)
	Coordinates_comment	Comment on how coordinates were imputed
Evidence type and methods	Evidence_type	Type of evidence the study provides: i. Empirical, quantitative ii. Empirical, qualitative iii. Theoretical iv. Suggestion anticipatory behaviours might occur, with explanation of mechanisms v. Reference to sources not screened in this systematic map (e.g. government reports, newspaper articles, documents in languages other than English, etc.)
	Secondary_evidence_source	Bibliographic details of source if evidence reported is contained in a source not screened in this systematic map
	Study_type	Study design type, or general approach of the study, e.g.: i. Quantitative impact evaluation ii. Ethnography iii. Policy impact analysis iv. Policy overview v. Historical overview vi. Economic modelling vii. Participatory mapping case study viii. Land change science analysis ix. Behavioural analysis x. Political ecology analysis xi. Randomized control trial policy impact evaluation xii. Review of ecological consequences of forest clearing xiii. Other
	Collection_methods	Data collection methods employed in the study, or source of data used in data analysis, e.g.: i. Interviews ii. Surveys iii. Focus group discussions iv. Satellite imagery processing v. Annual satellite data products processing vi. Cadastral coverage of properties vii. Regional newspapers viii. Policy documents ix. Census data x. Forest plot data xi. Parcel specific data xii. Certified plantations data xiii. Spatially-explicit property data xiv. Land conflict data xv. Government documents xvi. Participatory mapping workshops xvii. Field walks xviii. Timber market data xix. Agricultural prices data xx. Land clearing, deforestation or forest cover change data xxi. Fire data xxii. Case studies xxiii. Other
	Analysis_methods	Data analysis methods employed in the study, e.g.: i. Matching methods ii. Panel methods iii. Regression analysis iv. Cadastral data analysis v. Newspaper article review vi. Behavioural modelling vii. Predictive economic modelling viii. Qualitative analysis ix. Qualitative description x. Descriptive presentation of quantitative data xi. GIS data and satellite imagery analysis xii. Legal analysis xiii. Policy review xiv. Institutional review xv. Predictive theoretical analysis xvi. Hierarchical cluster analysis xvii. Principal component analysis xviii. Bayesian structural modelling xix. Statistical comparison of timber harvest rates xx. Hypothesis formulation based on case study review xxi. Theoretical framework elaboration xxii. Based on own empirical analysis (for theoretical type of evidence) xxiii. Other
Forest cover dynamics	Anticipatory_behaviour	Anticipatory behaviour reported as described by the authors
	Behaviour_objective	The objective of the anticipatory behaviour reported, according to authors of the study, e.g.: i. Subsistence agricultural expansion ii. Commercial agricultural expansion iii. Pasture expansion iv. Mining expansion v. Timber extraction / harvesting vi. Timber plantation expansion vii. Land development / urban expansion / settlement expansion viii. Charcoal / firewood production ix. Avoiding loss of entitlement to program x. Other xi. Not mentioned
	Post_policy_forest_dynamic	Post-policy implementation forest cover dynamic according to authors of the study, e.g.: i. Increased forest loss ii. Reduced forest loss iii. Increased forest degradation iv. Reduced forest degradation v. No difference found vi. Not mentioned
Policy context	Policy_type	The type of policy or policy tool that triggered the anticipatory behaviour, e.g.: i. Habitat conservation ii. Terrestrial protected area iii. Trading restrictions iv. Land registration programme v. Certification of agricultural commodities vi. Other
	Policy_name	Name of the policy that triggered the anticipatory behaviour
	Policy_dynamic	Whether the policy implemented is new, an amendment, update, etc.: i. New implementation ii. Policy amendment iii. Policy update iv. Provision within policy v. Conflict around existing policy vi. Voluntary adoption of policy vii. Other
	Policy_institution	Name of institution or organisation that introduced the policy
	Actors	Stakeholders, actors, and/or forest users deploying the anticipatory behaviour, according to authors’ description
Temporal dimension	Policy_year	When was the policy implemented (e.g. year)
	Policy_duration	How long the policy was in place (e.g. years, still in place, etc.)
	Behaviour_duration	How long the anticipatory behaviour lasted
Forest characteristics	Forest_type	Forest type as stated by the authors
	Forest_definition	Forest definition provided in the article, in the case it is provided
Link	Google_Scholar_link	Link to the article in Google Scholar

### Data synthesis and presentation

#### Narrative synthesis and presentation

We will summarize results through a narrative synthesis of the data provided by the included studies, supported by the following elements. We will present a ROSES flow diagram [21] detailing the systematic mapping process, including the number of articles obtained through each search strategy, those included and excluded at each screening stage, and the total number of relevant studies after full text level screening. We will provide a list of articles excluded after full text screening, with the reasons for exclusion. We will compute and present descriptive statistics on the distribution and trends of included studies. We will produce a world map presenting the location of the included studies, using the Shiny app EviAtlas [21], which allows to cluster points to aid visualization in the case the number of studies in a given region is too large. The world map will be provided in the final report of this systematic map and hosted online. Matrices or heatmaps presenting the number of studies for at least the following interactions between variables: type of intervention and anticipatory behaviour, country income level and anticipatory behaviour, and forest biome and anticipatory behaviour. In addition to the narrative report, world map, and accompanying figures and tables, we will upload the dataset online, with the possibility of filtering studies by for example, location, type of intervention, biome, or any other of the attributes extracted from the included studies (Table 4). The dataset will be free for users to download, which will be made available in a website we will create for this purpose, hosted in the first author’s GitHub account.

#### Knowledge gap and cluster identification strategy

We expect to be able to identify knowledge gaps on, for example, policy sectors, which might indicate where primary research is more urgently needed. Regarding knowledge clusters, given that this is the first evidence synthesis conducted on anticipatory forest use behaviours, we do not expect to be able to identify areas where the evidence base is large enough to be amenable to further, narrower systematic reviews. We will use all the knowledge gathered through this to elaborate hypotheses as of to why the evidence is more present or absent in certain regions or policies, and point to potential research and policy gaps accordingly.

#### Demonstrating procedural independence

No member of the review team will work on any articles authored by themselves, either at the screening or the data extraction stages.

## Supplementary information

**Additional file 1.** Initial article benchmark.

**Additional file 2.** Data extraction test.

**Additional file 3.** ROSES form for systematic map protocol.

**Additional file 4.** List of organisational websites for grey literature searches.

**Additional file 5.** Search string tests.

**Additional file 6.** Results from citation chasing test.

## Data Availability

All data generated or analysed during this study will be included in the published article and its supplementary information files.
