# Supplementary material for: Evidence of anticipatory forest use behaviours under policy introduction: a systematic map protocol
Source: Environ Evid. 2023 Sep 26;12:20. doi: 10.1186/s13750-023-00307-0 (PMC11116263; doi:10.1186/s13750-023-00307-0)
Supplement: Supplementary file 1 — Additional file 1. Initial article benchmark. [file 13750_2023_307_MOESM1_ESM.docx]

**Additional file 1: Initial article benchmark**

Below is a list of published studies relevant to this systematic map protocol. The list has been used to extract relevant terms to develop the search string.

1. Aldrich, S., R. Walker, C. Simmons, M. Caldas and S. Perz (2012). Contentious Land Change in the Amazon's Arc of Deforestation. *Annals of the Association of American Geographers* **102**(1): 103-128.
2. Alston, L. J., G. D. Libecap and B. Mueller (2000). Land Reform Policies, the Sources of Violent Conflict, and Implications for Deforestation in the Brazilian Amazon. *Journal of Environmental Economics and Management* **39**(2): 162-188.
3. Carlson, K. M., R. Heilmayr, H. K. Gibbs, P. Noojipady, D. N. Burns, D. C. Morton, N. F. Walker, G. D. Paoli and C. Kremen (2018). Effect of oil palm sustainability certification on deforestation and fire in Indonesia. *Proceedings of the National Academy of Sciences* **115**(1): 121-126.
4. Fiorini, A. C. O., M. Swisher and F. E. Putz (2020). Payment for Environment Services to Promote Compliance with Brazil’s Forest Code: The Case of “Produtores de Água e Floresta”. *Sustainability* **12**(19): 8138.
5. Grimsditch, M. and L. Schoenberger (2015). *New Actions and Existing Policies: The Implementation and Impacts of Order 01*. Phnom Penh, Cambodia, The NGO Forum on Cambodia, Land and Livelihoods Program.
6. Keller, E. (2015). *Beyond the Lens of Conservation. Malagasy and Swiss Imaginations of One Another*. New York and Oxford, Berghahn Books. 244 pp.
7. List, J. A., M. Margolis and D. E. Osgood (2006). *Is the Endangered Species Act Endangering Species? Working Paper Series 12777*, National Bureau of Economic Research.
8. Llopis, J. C., P. C. Harimalala, R. Bär, A. Heinimann, Z. H. Rabemananjara and J. G. Zaehringer (2019). Effects of protected area establishment and cash crop price dynamics on land use transitions 1990–2017 in north-eastern Madagascar. *Journal of Land Use Science* **14**(1): 52-80.
9. Lueck, D. and J. A. Michael (2003). Preemptive Habitat Destruction under the Endangered Species Act. *The Journal of Law and Economics* **46**(1): 27-60.
10. Mcgrath, C. (2007). End of Broadscale Clearing in Queensland. *Environmental and Planning Law Journal* **24**(1): 5-13.
11. Middleton, K. (2013). *Land Rights and Alien Plants in Dryland Madagascar*. Contest for Land in Madagascar. S. Evers, G. Campbell and M. Lambek. Leiden, Brill**:** 141-170.
12. Milne, S. (2013). Under the leopard's skin: Land commodification and the dilemmas of Indigenous communal title in upland Cambodia. *Asia Pacific Viewpoint* **54**(3): 323-339.
13. Nieminen, E., K. Salovaara, P. Halme and J. S. Kotiaho (2021). No evidence of systematic pre-emptive loggings after notifying landowners of their lands' conservation potential. *Ambio* **50**(2): 465-474.
14. Probst, B., A. BenYishay, A. Kontoleon and T. N. P. dos Reis (2020). Impacts of a large-scale titling initiative on deforestation in the Brazilian Amazon. *Nature Sustainability* **3**(12): 1019-1026.
15. Reside, A. E., J. Beher, A. J. Cosgrove, M. C. Evans, L. Seabrook, J. L. Silcock, A. S. Wenger and M. Maron (2017). Ecological consequences of land clearing and policy reform in Queensland. *Pacific Conservation Biology* **23**(3): 219-230.
16. Seghezzo, L., J. N. Volante, J. M. Paruelo, D. J. Somma, E. C. Buliubasich, H. E. Rodríguez, S. Gagnon and M. Hufty (2011). Native Forests and Agriculture in Salta (Argentina): Conflicting Visions of Development. *The Journal of Environment & Development* **20**(3): 251-277.
17. Simmons, B. A., E. A. Law, R. Marcos-Martinez, B. A. Bryan, C. McAlpine and K. A. Wilson (2018a). Spatial and temporal patterns of land clearing during policy change. *Land Use Policy* **75**: 399-410.
18. Simmons, B. A., R. Marcos-Martinez, E. A. Law, B. A. Bryan and K. A. Wilson (2018b). Frequent policy uncertainty can negate the benefits of forest conservation policy. *Environmental Science & Policy* **89**: 401-411.
19. Simmons, B. A., K. A. Wilson, R. Marcos-Martinez, B. A. Bryan, O. Holland and E. A. Law (2018c). Effectiveness of regulatory policy in curbing deforestation in a biodiversity hotspot. *Environmental Research Letters* **13**(12): 124003.
20. Stroup, R. L. (1995). *The Endangered Species Act: making innocent species the enemy. PERC Policy Series*.
21. Taylor, M. (2015). *WWF Briefing – Bushland destruction rapidly increasing in Queensland*, WWF-Australia.
22. Tomich, T. P. and M. Van Noordwijk (1995). What drives deforestation in Sumatra? *Paper presented at the Regional Symposium on “Montane Mainland Southeast Asia in Transition” Chiang Mai, Thailand, 13-16 November 1995.* Chiang Mai, Thailand.
23. Wren-Lewis, L., L. Becerra-Valbuena and K. Houngbedji (2020). Formalizing land rights can reduce forest loss: Experimental evidence from Benin. *Science Advances* **6**(26): eabb6914.
24. Zhang, D. (2004). Endangered species and timber harvesting: the case of red-cockaded woodpeckers. *Econ. Inquiry* **42**: 150–165.
