# Supplementary material for: Evidence of anticipatory forest use behaviours under policy introduction: a systematic map protocol
Source: Environ Evid. 2023 Sep 26;12:20. doi: 10.1186/s13750-023-00307-0 (PMC11116263; doi:10.1186/s13750-023-00307-0)
Supplement: Supplementary file 5 — Additional file 5. Search string tests. [file 13750_2023_307_MOESM5_ESM.docx]

**Additional file 3: Search string tests**

We conducted the following search tests in Web of Science Core Collection using the “Topic” option on 28th June 2023, using the subscription of Bangor University.

**First search string test**

We elaborated the first search string with the aim of capturing as many of the articles in the initial benchmark listed in Web of Science (15 out of 24 articles in the benchmark) as possible. For doing so, we required the results to include all research question elements, i.e. population, outcome (both forest use behaviour and character of behaviour) and intervention. We included the most common terms used to describe anticipatory forest use behaviours and synonyms, plus words explicitly employed by the articles in the initial benchmark for concrete policy interventions.

For the test, we attempted to reduce the results returned, by making explicit that both the character of the behaviour *and* the policy that triggered the anticipatory behaviour are captured:

(tree* OR *forest* OR woodland* OR "wood*-land" OR habitat* OR "vegetation" OR "timber" OR "canopy" OR "plant" OR "plants")

AND

(deforest* OR "clearing" OR "clearance" OR "clear" OR "cleared" OR destruct* OR extracti* OR "conversion" OR convert* OR destroy* OR "vanishing" OR fell* OR cut* OR "expansion" OR expand* OR "land use change" OR "land-use change" OR "land cover change" OR "land-cover change" OR "land change" OR "land-change" OR replac* OR remov* OR harvest* OR "log" OR "logging" OR "logged" OR exploit* OR "land management")

AND

(pre-empt* OR preempt* OR anticipat* OR prematur* OR "contentious" OR "contended" OR "unprecedented" OR "panic" OR exacerbat* OR accelerat* OR reinforc* OR rush* OR "unplanned" OR unpredict* OR unexpect* OR expectation* OR atypical* OR perverse* OR "unintended" OR spik* OR stimulat* OR "preventive" OR "preventative" OR paradox* OR undesir* OR violen* OR *incentiv* OR "ambiguous")

AND

(certif* OR "protected area*" OR "land reform" OR "forest law" OR "forest code" OR legislat* OR "titling" OR "title" OR "titled" OR "listing" OR "act" OR "protection program" OR "policy reform" OR "conservation policy")

Even though this string returned a manageable number of results, 3,355, it only captured 12 out of the 15 benchmark articles.

**Second search string test**

The second search string was developed with the aim of increasing the number of benchmark articles captured. For this, we refined the string in such a way that, while the population and the outcome-forest use behaviour elements are always captured, only either the character of the behaviour or the policy are captured. For this, we used the Boolean operator OR between the two latter elements, given that in some benchmark articles (e.g. Carlson *et al.* 2018), the anticipatory behaviour is not mentioned explicitly in title, abstract or keywords, although the policy is mentioned. Below is the second search string:

(tree* OR *forest* OR woodland* OR "wood*-land" OR habitat* OR "vegetation" OR "timber" OR "canopy" OR "plant" OR "plants")

AND

(deforest* OR "clearing" OR "clearance" OR "clear" OR "cleared" OR destruct* OR extracti* OR "conversion" OR convert* OR destroy* OR "vanishing" OR fell* OR cut* OR "expansion" OR expand* OR "land use change" OR "land-use change" OR "land cover change" OR "land-cover change" OR "land change" OR "land-change" OR replac* OR remov* OR harvest* OR "log" OR "logging" OR "logged" OR exploit* OR "land management")

AND

((pre-empt* OR preempt* OR anticipat* OR prematur* OR "contentious" OR "contended" OR "unprecedented" OR "panic" OR exacerbat* OR accelerat* OR reinforc* OR rush* OR "unplanned" OR unpredict* OR unexpect* OR expectation* OR atypical* OR perverse* OR "unintended" OR spik* OR stimulat* OR "preventive" OR "preventative" OR paradox* OR undesir* OR violen* OR *incentiv* OR "ambiguous")

OR

(certif* OR "protected area*" OR "land reform" OR "forest law" OR "forest code" OR legislat* OR "titling" OR "title" OR "titled" OR "listing" OR "act" OR "protection program" OR "policy reform" OR "conservation policy"))

This string managed to capture all 15 benchmark articles in Web of Science, although at the expense of returning 86,953 results, a number way higher than the review team could handle.

**Third search string test**

The third search string was elaborated with the aim of continue capturing as many benchmark articles as possible, but also reducing the number of results returned. For this, we employed the proximity operator NEAR/10 between the outcome-forest use behaviour and the outcome-character of behaviour elements, given that in most occasions both terms are expressed in proximity in the title or abstract of the benchmark articles. We also kept the condition that the population and the outcome-forest use behaviour elements are always captured, while only either the outcome-character of behaviour or the intervention elements are included, in the following way:

(tree* OR *forest* OR woodland* OR "wood*-land" OR habitat* OR "vegetation" OR "timber" OR "canopy" OR "plant" OR "plants")

AND

(((deforest* OR "clearing" OR "clearance" OR "clear" OR "cleared" OR destruct* OR extracti* OR "conversion" OR convert* OR destroy* OR "vanishing" OR fell* OR cut* OR "expansion" OR expand* OR "land use change" OR "land-use change" OR "land cover change" OR "land-cover change" OR "land change" OR "land-change" OR replac* OR remov* OR harvest* OR "log" OR "logging" OR "logged" OR exploit* OR "land management")

NEAR/10

(pre-empt* OR preempt* OR anticipat* OR prematur* OR "contentious" OR "contended" OR "unprecedented" OR "panic" OR exacerbat* OR accelerat* OR reinforc* OR rush* OR "unplanned" OR unpredict* OR unexpect* OR expectation* OR atypical* OR perverse* OR "unintended" OR spik* OR stimulat* OR "preventive" OR "preventative" OR paradox* OR undesir* OR violen* OR *incentiv* OR "ambiguous"))

OR

((deforest* OR "clearing" OR "clearance" OR "clear" OR "cleared" OR destruct* OR extracti* OR "conversion" OR convert* OR destroy* OR "vanishing" OR fell* OR cut* OR "expansion" OR expand* OR "land use change" OR "land-use change" OR "land cover change" OR "land-cover change" OR "land change" OR "land-change" OR replac* OR remov* OR harvest* OR "log" OR "logging" OR "logged" OR exploit* OR "land management")

AND

(certif* OR "protected area*" OR "land reform" OR "forest law" OR "forest code" OR legislat* OR "titling" OR "title" OR "titled" OR "listing" OR "act" OR "protection program" OR "policy reform" OR "conservation policy")))

This string still captured all benchmark articles appearing in Web of Science, but still returned 37,015 articles, more than feasible for the review team to screen.

**Fourth search string test**

For the fourth search string test, we removed some terms that were too general, to attempt to reduce the number of results obtained. We concretely removed “plant” and “plants” from the population element, and “act” from the intervention element.

(tree* OR *forest* OR woodland* OR "wood*-land" OR habitat* OR "vegetation" OR "timber" OR "canopy")

AND

(((deforest* OR "clearing" OR "clearance" OR "clear" OR "cleared" OR destruct* OR extracti* OR "conversion" OR convert* OR destroy* OR "vanishing" OR fell* OR cut* OR "expansion" OR expand* OR "land use change" OR "land-use change" OR "land cover change" OR "land-cover change" OR "land change" OR "land-change" OR replac* OR remov* OR harvest* OR "log" OR "logging" OR "logged" OR exploit* OR "land management")

NEAR/10

(pre-empt* OR preempt* OR anticipat* OR prematur* OR "contentious" OR "contended" OR "unprecedented" OR "panic" OR exacerbat* OR accelerat* OR reinforc* OR rush* OR "unplanned" OR unpredict* OR unexpect* OR expectation* OR atypical* OR perverse* OR "unintended" OR spik* OR stimulat* OR "preventive" OR "preventative" OR paradox* OR undesir* OR violen* OR *incentiv* OR "ambiguous"))

OR

((deforest* OR "clearing" OR "clearance" OR "clear" OR "cleared" OR destruct* OR extracti* OR "conversion" OR convert* OR destroy* OR "vanishing" OR fell* OR cut* OR "expansion" OR expand* OR "land use change" OR "land-use change" OR "land cover change" OR "land-cover change" OR "land change" OR "land-change" OR replac* OR remov* OR harvest* OR "log" OR "logging" OR "logged" OR exploit* OR "land management")

AND

(certif* OR "protected area*" OR "land reform" OR "forest law" OR "forest code" OR legislat* OR "titling" OR "title" OR "titled" OR "listing" OR "protection program" OR "policy reform" OR "conservation policy")))

This search string managed to capture the 15 benchmark articles listed in Web of Science, but still returned 19,290 results, beyond the screening capacity of the review team.

**Fifth search string test**

For the fifth search string test, we removed the intervention element from the search string, in the hope to reduce the number of returned articles. We took this decision given that, after careful examination of the articles being returned by the previous search string, we realised that we were obtaining many results irrelevant to the objective of this systematic map. This was largely due to the inclusion of policy terms without corresponding terms for anticipatory forest use behaviours. Below is the fifth search string:

(tree* OR *forest* OR woodland* OR "wood*-land" OR habitat* OR "vegetation" OR "timber" OR "canopy")

AND

((deforest* OR "clearing" OR "clearance" OR "clear" OR "cleared" OR destruct* OR extracti* OR "conversion" OR convert* OR destroy* OR "vanishing" OR fell* OR cut* OR "expansion" OR expand* OR "land use change" OR "land-use change" OR "land cover change" OR "land-cover change" OR "land change" OR "land-change" OR replac* OR remov* OR harvest* OR "log" OR "logging" OR "logged" OR exploit* OR "land management")

NEAR/10

(pre-empt* OR preempt* OR anticipat* OR prematur* OR "contentious" OR "contended" OR "unprecedented" OR "panic" OR exacerbat* OR accelerat* OR reinforc* OR rush* OR "unplanned" OR unpredict* OR unexpect* OR expectation* OR atypical* OR perverse* OR "unintended" OR spik* OR stimulat* OR "preventive" OR "preventative" OR paradox* OR undesir* OR violen* OR *incentiv* OR "ambiguous"))

This search string reduced the results returned to 7,158 results, which is within the limits of what the review team can aim at screening, although at the expense of also reducing the number of benchmark articles captured to 12 out of 15 articles.

**Sixth search string test**

Our last attempt tried to compensate for not being able to capture more benchmark articles, by slightly expanding the scope of the search including more terms in the population element of the research question, concretely mangrove*, savanna*, “cerrado” and “caatinga”. Further, we also tightened the outcome-forest use behaviour element, using the wildcard character (*) instead of alternative uses of several terms, concretely for vanish*, logg* and clear*, while leaving the outcome-character of behaviour element unchanged:

(tree* OR *forest* OR woodland* OR “wood*-land” OR habitat* OR vegetation OR timber OR canopy OR mangrove* OR savanna* OR “cerrado” OR “caatinga”)

AND

((deforest* OR clear* OR destruct* OR extracti* OR "conversion" OR convert* OR destroy* OR vanish* OR fell* OR cut* OR “expansion” OR expand* OR "land use change" OR "land-use change" OR "land cover change" OR "land-cover change" OR "land change" OR "land-change" OR replac* OR remov* OR harvest* OR logg* OR exploit* OR “land management”)

NEAR/10

(pre-empt* OR preempt* OR anticipat* OR prematur* OR "contentious" OR "contended" OR “unprecedented” OR “panic” OR exacerbat* OR accelerat* OR reinforc* OR rush* OR “unplanned” OR unpredict* OR unexpect* OR expectation* OR atypical* OR perverse* OR “unintended” OR spik* OR stimulat* OR "preventive" OR "preventative" OR paradox* OR undesir* OR violen* OR *incentiv* OR “ambiguous”))

The final test search string increased the results to 7,431 records, although capturing the same number of benchmark articles (12) out of those listed in Web of Science (15) than the previous test. After assessing the comprehensiveness of our overall search approach (e.g. including the citation chase strategy), we consider this number satisfactory and we decided to settle on this search string for our bibliographic searches.
